# Effect of the molecular targeted drug, erlotinib, against endometrial cancer expressing high levels of epidermal growth factor receptor

**DOI:** 10.1186/s12885-015-1975-5

**Published:** 2015-12-16

**Authors:** Toshio Nishimura, Kazuto Nakamura, Soichi Yamashita, Sadatomo Ikeda, Keiko Kigure, Takashi Minegishi

**Affiliations:** Department of Obstetrics and Gynecology, Gunma University, 3-39-22, Showa, Maebashi, Gunma 371-8511 Japan; Gunma Prefectural Cancer Center, 617-1, Nishimachi, Takabayashi, Ota, Gunma 373-8500 Japan

**Keywords:** Molecular targeted drug, Erlotinib, Endometrial cancer, EGFR

## Abstract

**Background:**

The epidermal growth factor receptor (EGFR) tyrosine kinase inhibitor, erlotinib, has been clinically applied for the treatment of a variety of tumors with EGFR overexpression. A phase II clinical study of erlotinib (NCIC IND-148) for recurrent or metastatic endometrial carcinoma (EC) resulted in an unfavorable result. However, in that study, the expression levels of EGFR were not accurately analyzed. Thus, the aim of this study was to re-examine the efficacy of erlotinib in EC cells by utilizing in vitro and in vivo models.

**Methods:**

Tissue samples obtained from patients histologically diagnosed with EC of the uterine corpus were subjected to immunohistochemistry and RT-PCR to determine the protein and mRNA expression levels of EGFR. Western blot and WST-1 assays of EGFR siRNA-transfected HEC-1A, KLE, and Ishikawa cells were used to evaluate the efficacy of erlotinib in tumor cell lines expressing different EGFR levels. Furthermore, HEC-1A and Ishikawa cells were implanted into athymic mice treated with either erlotinib or trastuzumab.

**Results:**

At our institution, 20.9 % of endometrial cancer patients with low grade endometrioid histology have been diagnosed as stage III and IV. Immunohistochemical analysis and RT-PCR revealed the presence of significant EGFR and EGFR mRNA expression in low-grade endometrioid carcinoma in comparison with high-grade endometrioid carcinoma. In vitro study, WST-1 assay and Western blot analysis revealed that EGFR expression levels were correlated with tumor cell viability. Erlotinib reduced the proliferation of HEC-1A expressing high levels of EGFR, while trastuzumab showed similar effect in Ishikawa cells dominantly expressing human epidermal growth factor receptor type2 (HER2). In vivo erlotinib decreased tumor growth in mice xenografted with HEC-1A cells, whereas this tumor-growth inhibition was not observed in trastuzumab-treated mice xenografted with Ishikawa cell.

**Conclusions:**

EGF contributed to tumor proliferation in EC cell lines along with EGFR expression in vitro. Erlotinib also demonstrated anti-tumor effects in xenograft mice models. Our results suggest that erlotinib continues to have clinical usefulness in specific cases, after taking into consideration the EGFR expression levels.

## Background

Endometrial carcinoma (EC) is one of the most common gynecological malignant tumors in Japan; over 8000 women were diagnosed with it in 2012. There are two subtypes of endometrial carcinoma according to the clinico-pathological characteristics: type I EC and type II EC [1, 2]. Recent studies on gene signature in EC cells have reported numerous genetic disorders that initiate carcinogenesis: for instance, PTEN, which regulates normal cell function, is highly mutated in type I EC, whereas p53, which prevents genome mutation, is altered by up to 80–90 % in type II EC [3].

Type I EC accounts for about 80 % of EC, and is generally associated with better prognosis than type II EC since it is composed of low grade endometrioid histology with less aggressive characteristics and favorable prognosis [3]. However, the number of patients with advanced stage or recurrent low-grade tumors might not be negligible since type I EC comprises about 80 % of the newly diagnosed EC in Western Europe, North America, and Japan [3, 4].

After staging surgery, adjuvant therapy is considered based on the pathological risk factors, such as tumor grade, histological type, myometrial invasion, positive margin, lymphovascular space invasion, and positive node status [5]. Radiotherapy has proved to reduce the risk of local recurrence, but no randomized study has shown benefit for overall survival [6, 7]. In the last decades, there has been emerging evidence suggesting that systemic cytotoxic chemotherapy may have favorable prognosis in advanced EC [8, 9]. Taxanes, platinum agents, and anthracyclines have been utilized in advanced and recurrent EC patients, with response rates to these drugs ranging from 33 to 57 % [8, 10–14].

Cytotoxic cancer therapy induces apoptosis in cancer cells by inhibiting microtubule function, protein function, or DNA synthesis. In contrast, molecular-targeted therapy is designed to interfere with specific molecules involved in cancer cell proliferation and metastasis. Recently, a better understanding of the molecular and genetic characteristics of EC has promoted clinical research that targets angiogenesis and cellular signaling pathways involved in cancer development and progression. Epidermal growth factor receptor (EGFR) has been shown to be overexpressed in many human cancers, including lung [15, 16], central nervous system [17], head and neck [18], bladder [19], pancreas [20], and breast [21]; and it correlates with poor prognosis [22]. EGFR expression has been found to be associated with patient outcomes in 43–67 % of EC tissue samples [23–25] and different profiles in low and high grade EC [26]. Furthermore, some studies have reported that the EGFR family plays an important role in the development of EC [27, 28]. Collectively, inhibition of EGFR function can be a clinical benefit. Targeted therapy against the signaling system of the tyrosine kinase family could be beneficial for patients with type II EC [29, 30]. Those reports found that the expression of EGFR and human epidermal growth factor receptor type2 (HER2) in type II EC was 30.5 and 16.5 %, respectively, whereas it was 51.5 and 2 % in well (Grade 1, G1) and moderately (G2) differentiated endometrioid cancer, respectively. However, there have been no promising therapies, including small molecule tyrosine kinase inhibitors or anti-EGFR monoclonal antibodies, for antagonizing EGFR functions [31, 32]. Thus, in this study, we aimed to evaluate whether targeting the EGFR tyrosine kinase may have a therapeutic effect against EC, by accurately assessing the expression levels of EGFR in cancer cells.

## Methods

### Reagents

Erlotinib (Abcam, Tokyo, Japan) was dissolved in DMSO, and pertuzumab (Tyugai, Tokyo, Japan) was dissolved in distilled water for the in vitro and in vivo studies. Epidermal growth factor (EGF) (Invitrogen, Carlsbad, CA, USA) was dissolved in phosphate buffered saline (PBS) (stock solution: 20 ng/mL).

DMEM (without phenol red) and gentamicin sulfate (Geneticin) were purchased from Invitrogen (Carlsbad, CA, USA). DMEM/Ham’s nutrient mixture F-12 (1:1, vol/vol) (without phenol red) was purchased from Sigma-Aldrich (St. Louis, MO, USA).

### Cell culture and culture condition

Ishikawa cells were purchased from Japanese Collection of Research Bioresources (JCRB) cell bank (Tokyo, Japan). HEC293, HEC-1A, and KLE cells were purchased from American Type Culture Collection (Manassas, VA, USA). All the cells used for the experiments were between the 5^th^ and 20^th^ passages.

HEC293 and Ishikawa cells were maintained in DMEM supplemented with 10 % charcoal fetal bovine serum (FBS) and 50 μg/μL gentamicin sulfate. HEC-1A cells were maintained in McCoy’s 5A medium supplemented with 10 % charcoal FBS. KLE cells were maintained in DMEM/nutrient mix F-12 Ham’s supplemented with 5 % charcoal FBS. All media used were phenol red free. Cells were incubated at 37 °C in a humidified atmosphere containing 5 % CO_2_. All cells were harvested using trypsin/EDTA when confluence was less than 80 %. All cells were used all experiments when cell concentration was 60–70 %.

### Tissues and patient cohort

Resected tissue samples (only hysterectomy samples without neoadjuvant treatment) were obtained from 51 endometrioid adenocarcinoma patients of Asian descent between May 2007 and March 2011 from Gunma University Hospital. All patients had been identified as uterine corpus endometrioid adenocarcinoma carriers on the basis of gynecologic and pathological reports. Staging was defined according to the International Federation of Obstetricians and Gynecologists (FIGO) Surgical staging system [33]. The histological grade classification was according to the World Health Organization classification and FIGO [34]. This study was approved by the Institutional Review Board of Gunma University (Permit Number: 12–49) and conducted according to the ethical guidelines of Gunma University. Tissue specimens were handled according to the local ethics committee guidelines. Written informed consent was obtained from all patients enrolled in the study.

### Immunohistochemistry

Formalin fixed samples were embedded in paraffin and sectioned and dried; then, they were deparaffinized and rehydrated. The sections were immunostained using DAKO ENVISION+ KIT/HRP (DAKO, Carpentaria, CA, USA) and Histofine SAB-PO kit (Nichirei, Tokyo, Japan) according to manufacturers’ protocols. Rabbit monoclonal anti-EGFR antibody (diluted 1:100, DAKO, Carpentaria, CA, USA) and mouse monoclonal anti-HER-2 antibody (Santa Cruz Biotechnology, Santa Cruz, CA, USA) were used for immunohistochemistry (IHC) to determine EGFR and HER-2 expression levels. Two independent pathologists who were blinded to the clinical information assessed EGFR and HER-2 expression. The inter-observer disagreements (kappa statistics: 0.91) were reviewed a second time, followed by a conclusive decision by both pathologists. A positive result was defined as cell membrane staining of 50 % or above, independently of intensity.

### Western blotting

Twenty-four hours before starting the analysis, all cells were changed to a free- FBS medium. For the analysis of phosphorylated extracellular signal-regulated kinases (ERK) 1/2, cells were treated with EGF (range from 1 pg/mL to 1 ng/mL) for 10 min, washed twice with cold PBS, and incubated on ice with RIPA buffer (pH 7.4, supplemented with protease inhibitors, 200 mM NaF, 200 mM sodium orthovanadate) for 30 min. Lysates were aspirated and centrifuged at 15,000 rpm for 10 min at 4 °C. The protein concentration was measured in the collected supernatant. Frozen patient samples were homogenized and lysed in RIPA buffer. Protein samples (10–20 mg) were diluted in equal volume sample buffer (pH 6.8, 4 % SDS, 10 % 2-mercaptoethanol, 20 % glycerol, 0.004 % bromophenol blue, 0.125 M Tris–HCl) and incubated for 30 min at 25 °C. Protein samples were loaded on a 12 % polyacrylamide/bisacrylamide SDS-PAGE gel and transferred onto a PVDF membrane (BIO-RAD, Hercules, CA, USA). Membranes were blocked with 5 % BSA or 5 % skim milk in TBST (100 mM Tris, 0.9 % NaCl, 0.1 % Tween-20, pH 7.4) for 1 h at room temperature. Membranes were incubated overnight at 4 °C with the primary antibody (phosphor-ERK 1/2 at 1:2000, total-ERK at 1:1000, EGFR at 1:1500, rabbit anti-human HER-2 at 1:1000 [Cell Signaling Technology, MA, USA], and mouse anti-human beta-actin at 1:3000 [Sigma-Aldrich]). After incubation, the membranes were washed 5 times with TBST and incubated with the appropriate secondary antibody conjugated to horseradish peroxidase (anti-rabbit or mouse at 1:40000, BIO-RAD) for 1 h at room temperature. After washing 5 more times with TBST, the membranes were incubated with Immobilon Western Detection reagent (Millipore, Billerica, MA, USA) for 5 min and detected by an Image Quant Imager (GE Healthcare Bio Science). The expression levels of phosphorylated ERK were quantified by scanning the digital image and digitized data were analyzed with the Image J (NIH, USA).

### RNA isolation and quantitative RT-PCR

RNA was extracted from the endometrial cancer cell lines and primary resected endometrioid adenocarcinoma tissue samples. Total cellular and tissue RNA were extracted using Isogen (WAKO, Osaka, Japan) and 2 μg total RNA was treated with DNase I (Isogen, De Meern, Netherlands) according to manufacturer’s protocol. RNA was reverse transcribed using SuperScript III transcriptase (Invitrogen) with random primers (Invitrogen). The samples were incubated with RNAse at 37 °C to remove RNA, and were diluted with distilled water for a final volume of 100 μL. Each quantitative PCR consisted of 5 μL of cDNA template, 12.5 μL SYBR Green real-time PCR master mix (Toyobo, Osaka, Japan), 0.2 μL forward and reverse primers (50 μM), and 7.1 μL distilled water. The sequences for the forward and reverse primers are as follows: human EGFR: 5’ –GGAGAACTGCCAGAAACTGACC- 3’ and 5’ –GCCTGCAGCACACTGGTTG- 3’; human HER-2: 5’ –ATCTGGCGCTTTTGGCACAG- 3’ and 5’ –CACCAGCCATCACGTATGCT- 3’; human GAPDH: 5’ -AATTCCATGGCACCGTCAAG- 3’ and 5’ –GGTGAAGACGCCAGTGGACT- 3’. The reactions were carried out in an ABI PRISM 7000 sequence detection system (Applied Biosystems, Foster City, CA, USA) for 40 cycles (95 °C for 15 s, 60 °C for 1 min) after 1 min initial incubation at 95 °C. The fold change in the expression levels of each gene was calculated using the standard curve method, with GAPDH as an internal control.

### siRNA transfection

SiRNA against human EGFR (EGFR siRNA) or HER-2 (HER-2 siRNA), and siRNA for negative control (control siRNA) were obtained from Applied Biosystems. All cell lines were plated for 24 h to approximately 50 % confluence, and were transfected with 10 nM siRNA using Lipofectamine RNAiMAX (Ambion, Grand Island, NY, USA). The transfected cells were subjected to western blotting, quantitative RT-PCR, and growth inhibition assay.

### Growth inhibition assay

Cells were plated at a concentrations of 5000 cells (Ishikawa, KLE) or 10,000 cells (HEC-1A) per well in 96-well plates. After 12 h incubation at 37 °C in a humidified atmosphere containing 5 % CO_2_, the cells were treated with the pharmacological compounds (ErbB inhibitor: erlotinib [EGFR tyrosine kinase inhibitor) and trastuzumab [HER-2 monoclonal antibody]) or transfected with siRNA, and incubated for further 48 h in the same conditions. Erlotinib was dissolved in DMSO and added to the cell culture medium at a concentration not exceeding 0.1 % (v/v). At the end of various treatments, 10 μL cell counting solution (WST-1, Dojindo Labs, Tokyo, Japan) was added. Cells were incubated for 1 h and the absorbance was measured at a wavelength of 450–650 nm using a Microtiter Plate Reader (Becton Dickinson, Franklin Lakes, NJ, USA).

### Tumor xenograft model and treatment

All procedures involving animals followed ethical principles according the NIH Guide for Care and Use of the Laboratory Animals and were approved by the Gunma University Animal Care and Use Committee (Permit Number: 13–042). Female mice, 4-weeks-old nude BALB/C nu/nu, were obtained from Charles River Japan (Tokyo, Japan). Mice were housed in suitable cages in a pathogen-free condition in a room maintained at 23–26 °C, 50 % humidity, and 12-h light/12-h dark cycle. The mice were allowed to acclimatize for 2 weeks prior to the study. Regular health checks were performed. Mice were implanted with tumor cells at a single subcutaneous (s.c.) site on the shoulder flank (5 × 10^5^ HEC-1 and 1 × 10^6^ Ishikawa per mouse in a 0.1 mL growth factor reduced matrigel (Corning, Tewksbury, MA, USA) and 0.1 mL culture medium. Tumor-bearing mice were randomized into erlotinib (1 mg, 3 mg, 10 mg, 30 mg/kg/day, intraperitoneal (i.p.) for 5 days per week), pertuzumab (1 mg, 3 mg, 10 mg/kg, i.p. twice per week), and vehicle (DMSO and distilled water, i.p.) groups when the mean tumor volume was 100–150 mm^3^. Equal volume of the vehicle (0.1 mL) was injected in all animals. Tumor volume and body weight were determined twice weekly. The tumor volume was determined according to the following formula: tumor volume = (length) x (width)^2^/2. On day 28, mice were euthanized; tumor was excised, and fixed in formalin. Tumors were processed for hematoxylin and eosin (HE) staining.

### Data analysis

The data represent the mean ± standard error of the mean (SEM) from at least three independent experiments.

Comparisons between groups were performed by one-way ANOVA or chi-square test. The significance of the differences between the mean values of the control group and each treated group was determined by Dunnett’s multiple-comparison test. These analyses were 2-tailed tests, and a value of *P* < 0.05 was considered significant. The cumulative survival curve was estimated by the Kaplan-Meier method. All analyses were performed with IBM SPSS statistics 21 software.

## Results

### Expression of EGFR and HER-2 in endometrial cancer

**Fig. 1:**
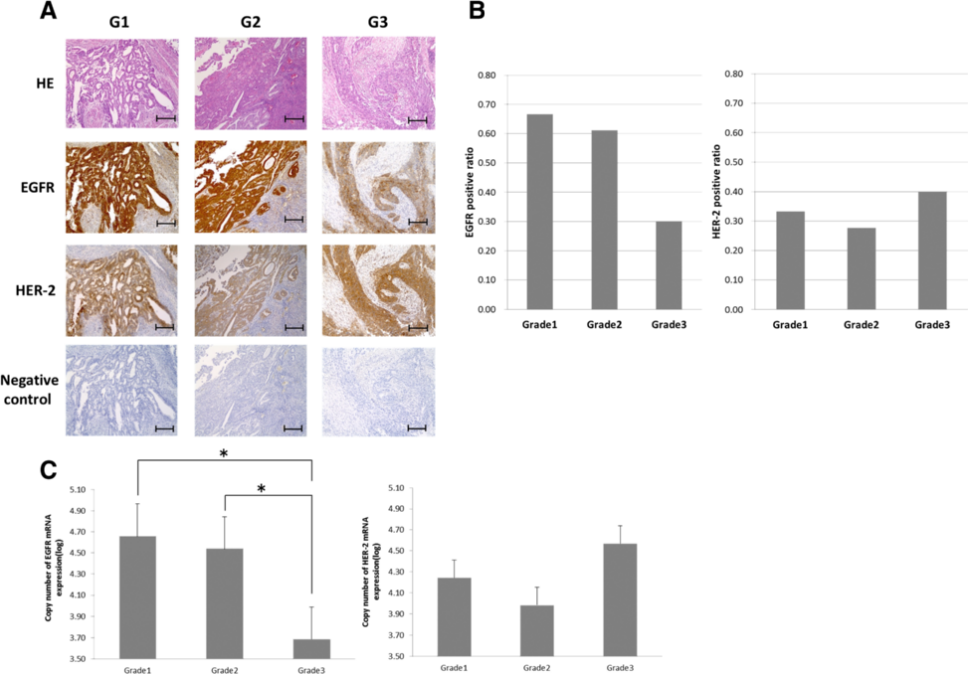
Detection of EGFR and HER-2 proteins in endometrial adenocarcinoma (surgically resected endometrioid cancer sample). **a** We used tissue samples of well differentiated (G1), moderately differentiated (G2), and poorly differentiated (G3) endometrial carcinoma for immunohistochemical study. The tissues were fixed in formalin and embedded in paraffin. Sections were taken from the paraffin-embedded tissue and stained with 1:200 anti-EGFR or 1:150 anti-HER-2. Primary antibody binding was detected through a biotin-conjugated secondary antibody. Top panels, HE stained; upper middle panels, stained with anti-EGFR; lower middle panels, stained with anti-HER-2; bottom panels, negative control. Magnification × 200. ars =1000 μm. **b** The expression status of EGFR and HER-2 in each grade of tumor were assessed by immunohistochemistry. The ratio of immunopositive cases for each protein is represented in the bars graph. **c** The carcinoma portions were excised, and RNA was isolated. EGFR and HER-2 mRNA levels were measured using quantitative RT-PCR. GAPDH mRNA levels were quantitated as an internal control. The amounts of EGFR and HER-2 mRNA were respectively normalized by the amounts of GAPDH mRNA. *, Decrease in the expression level of EGFR mRNA in G3 compared to those in G1 and G2 cancers, *P* < 0.05

Fifty-one surgically resected endometrioid carcinoma samples, classified as: well (Grade 1, G1), moderately (G2), or poorly (G3) differentiated adenocarcinoma, were obtained from patients who had undergone surgery at Gunma University Hospital (Table 1). In our institution, 20.9 % of patients with endometrial cancer with low-grade endometrioid histology were diagnosed as stage III and IV. As a first step, IHC was carried out on endometrial carcinoma to confirm the expression of EGFR and HER-2 proteins (Fig. 1a and b). EGFR protein was highly expressed in G1 and G2 endometrioid carcinoma (*p* = 0.014, *χ*^2^ (2) = 8.6) whereas HER-2 was almost evenly expressed in G1, G2, and G3 tumors (*P* = 0.52, *χ*^2^ (2) = 1.5). We also evaluated EGFR and HER-2 mRNA expression levels in EC tissues by RT-PCR (Fig. 1c). EGFR mRNA levels were higher in G1 and G2 (*P* < 0.05) than in G3, but there was no significant difference in HER-2 mRNA expression between the three grades.

**Table 1:** Characteristics of surgical cancer patients

	Well differentiated	Mediate differentiated	Poorly differentiated	*P* value
	(G1) (*n* = 4)	(G2) (*n* = 17)	(G3) (*n* = 10)	
Age^†^	57 (50–63)	59 (56–66)	58 (52–61)	ns
BMI^†^	22.2 (20.0–28.6)	23.4 (20.3–25.0)	21.4 (20.7–23.6)	ns
FIGO Surgical Stage				ns
I	16 (66.7 %)	10 (58.8 %)	5 (50.0 %)	
II	3 (12.5 %)	1 (5.9 %)	0 (0.0 %)	
III	4 (16.7 %)	6 (35.3 %)	3 (30.0 %)	
IV	1 (4.2 %)	0 (0 %)	2 (20.0 %)	

^†^Median (Interquartile Range). *FIGO* International Federation of Gynecology and Obstetrics

We did further Kaplan-Meier analysis of survival with patients who expressed or not EGFR by IHC. No significant differences were found between the two groups for overall survival (data not shown).

### EC cell line experiments

**Fig. 2:**
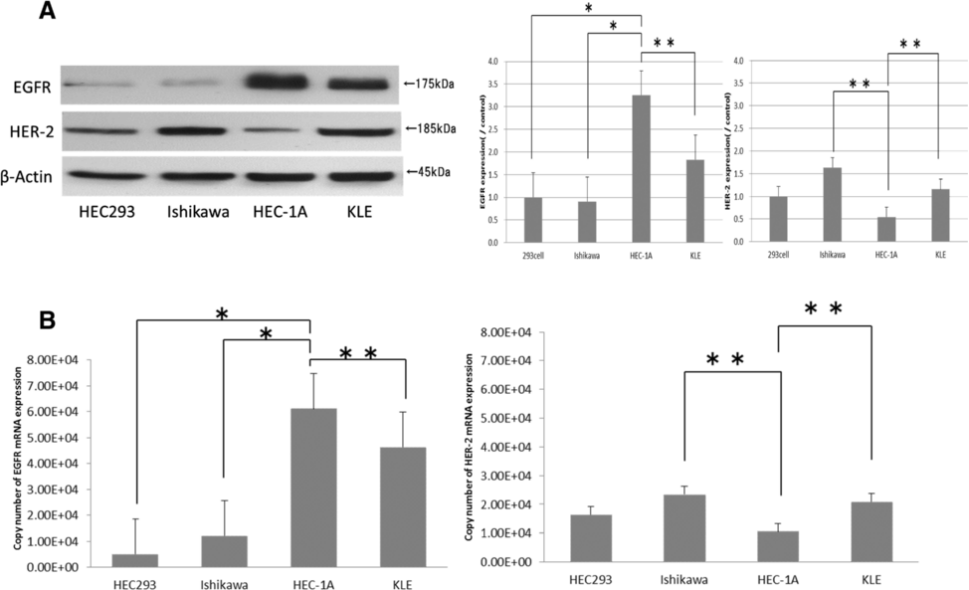
EGFR and HER-2 protein and mRNA expression levels in EC cell lines. **a** Cells were cultured, harvested, solubilized in detergent, and resolved by 12 % reducing SDS-PAGE. Each sample was confirmed with anti-EGFR, anti-HER-2, and anti-β-actin antibody. The detection of β-actin protein served as a loading control. The blot is representative of three independent experiments. *, increased expression levels of EGFR protein in HEC-1A compared to those in HEC293 and Ishikawa, *P* < 0.001 **, increased expression levels of EGFR protein in HEC-1A compared to those in KLE, and increased expression levels of HER-2 protein in Ishikawa and KLE compared to those in HEC-1A, *P* < 0.05. **b** EGFR and HER-2 mRNA levels were measured by quantitative RT-PCR. Data were normalized with GAPDH mRNA levels in each sample. Data represent the means ± SEMs of five independent experiments. *, increased expression levels of EGFR mRNA in HEC-1A compared to those in HEC293 and Ishikawa, *P* < 0.005 **, increased expression level of EGFR mRNA in HEC-1A compared to those in KLE, and increased expression level of HER-2 mRNA in Ishikawa and KLE compared to those in HEC-1A, *P* < 0.05

Cancer cell lines were utilized for further experiments to elucidate the roles of EGFR and HER-2 in EC cells. Three cell lines (Ishikawa, HEC-1A, and KLE) were evaluated by western blotting to determine protein expression levels of EGFR and HER-2. HEC-1A showed high EGFR and low HER-2 expression, while Ishikawa had low EGFR and high HER-2 expression. In KLE, the expression levels of EGFR and HER-2 were intermediate between Ishikawa and HEC-1A (Fig. 2a). These results were reconfirmed by quantitative RT-PCR experiments, which indicated that EGFR mRNA levels were significantly the highest in HEC-1A (*P* < 0.005), and HER-2 mRNA levels were highly expressed in Ishikawa (*P* < 0.05) (Fig. 2b).

**Fig. 3:**
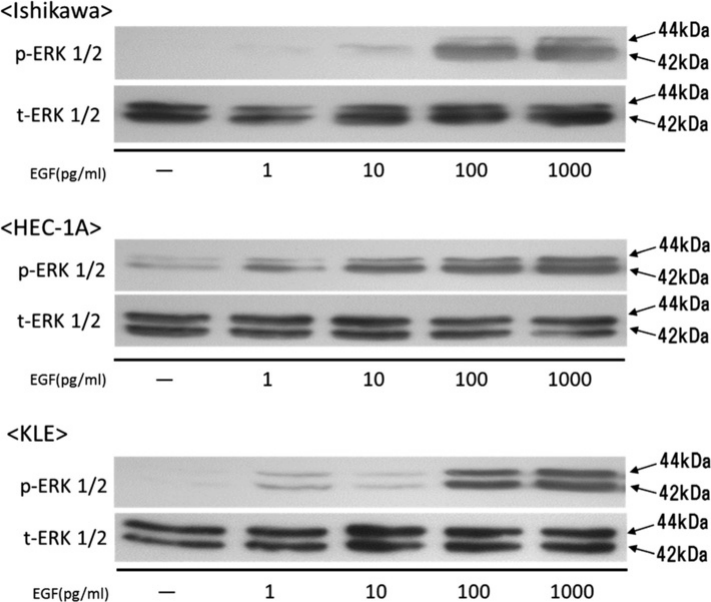
Phosphorylation of ERK treated EGF in EC cell lines. EC cells were incubated with epithelial growth factor (EGF) (1–1000 pg/mL), and cells were harvested at the 10 min for western blotting. Each sample was confirmed with either anti-phospho-ERK or anti-total-ERK

The three cell lines were treated with EGF and were evaluated for downstream signaling of EGFR, by detecting phosphorylated ERK 1/2 by western blotting (Fig. 3). The phosphorylation of ERK 1/2 was found to be induced in all three cell lines, but in HEC-1A, the increase occurred at a lower concentration compared to the other two cell lines. This result suggested that the amount of EGFR expression was an important factor for the activation of mitotic-activated protein kinase (MAPK) pathway by EGF stimulation in endometrial carcinoma cells.

**Fig. 4:**
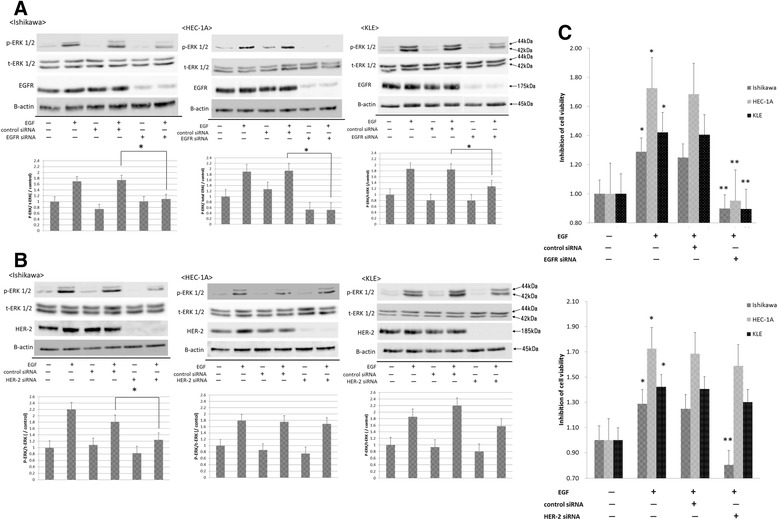
EGFR is involved in ERK phosphorylation in EC cell lines. All EC cells were transfected with 10 nM of siRNA (control, EGFR, or HER-2). Cells were harvested 48 h after transfection to evaluate ERK phosphorylation after knockdown of EGFR (**a**) or HER-2 protein (**b**). Cells were incubated with EGF (1 ng/mL) for 10 min and harvested for western blot analysis. The detection of β-actin protein served as a loading control. The blot is representative of three independent experiments. The expression levels of phosphorylated ERK were quantified by scanning the digital image and digitized data were analyzed with the Image J. Data represent the means ± SEMs of three independent experiments. *, decreased compared to siRNA control transfection (NC), *P* < 0.05. **c** All EC cells were transfected with 10 nM of siRNA (control, EGFR or HER-2), and cell proliferation was monitored after 48 h using WST-1 assay. *, increased compared to no treatment cell, *P* < 0.05.**, decreased compared to siRNA control transfection (Negative Control), *P* < 0.05

To investigate the significance of EGFR and HER-2 in the proliferation of endometrial cancer cells, all cells were transfected with siRNA to knock down EGFR or HER-2. After 48 h, EGF was added, and ERK 1/2 phosphorylation and proliferation were evaluated. When EGFR was knocked down (Fig. 4a and c), all cells showed decreased ERK 1/2 phosphorylation (*P* < 0.05). The viability of Ishikawa cells was reduced to 72 %, HEC-1A to 57 %, and KLE to 64 %, compared to the negative control (*P* < 0.05). When HER-2 was knocked down (Fig. 4b and c), ERK 1/2 phosphorylation was significantly decreased in Ishikawa, which highly expressed HER-2 (*P* < 0.05), but not in HEC-1A and KLE. Similarly, cell viability was reduced in Ishikawa (to 65 % compared with negative control) (*P* < 0.05), but not in other cell types (HEC-1A: to 94 % KLE: to 93 % compared with negative control).

### Growth inhibition assay following ErbB inhibitor treatment in vitro

**Fig. 5:**
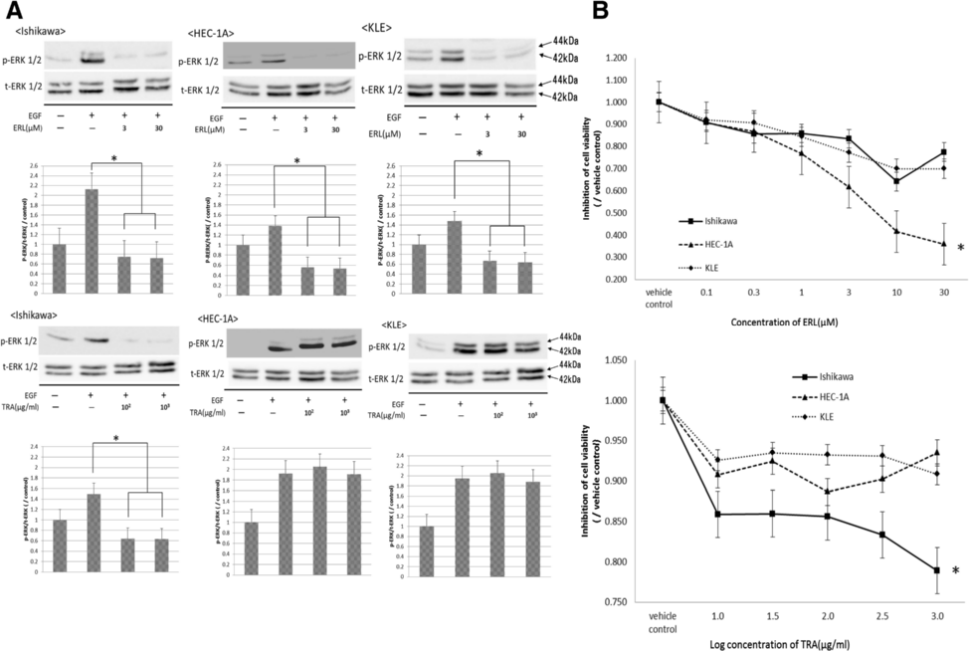
Effect of ERK phosphorylation by erlotinib or trastuzumab on proliferation in EC cell lines. **a** All cells were treated with either ERL (3 μM, 30 μM) or TRA (100 μg/mL, 1000 μg/mL). After a 2-h incubation with the drug, cells were treated EGF (1 ng/mL) for 10 min and harvested for western blot analysis. The blot is representative of three independent experiments. The expression levels of phosphorylated ERK were quantified by scanning the digital image and digitized data were analyzed with the Image J. Data represent the means ± SEMs of three independent experiments. *, decreased with the drug treatment compared to control, *P* < 0.001. **b** All cells were treated with either ERL (0.1–30 μM) or TRA (10–1000 μg/mL). After 2 h incubation with the drug, all cells were treated EGF (1 ng/mL). Cell proliferation was monitored after 96 h using WST-1 assay. *, decreased as compared to vehicle control, *P* < 0.01

The results in Fig. 4 prompted us to investigate whether ErbB inhibitors could effectively inhibit EC proliferation. In subsequent experiments, all cells were treated with erlotinib (ERL: EGFR tyrosine kinase inhibitor) or trastuzumab (TRA: HER-2 monoclonal antibody), and evaluated for ERK 1/2 phosphorylation and proliferation in EC cells. All cells treated with ERL showed decreased ERK 1/2 phosphorylation (*P* < 0.001) (Fig. 5a) and reduction in cell viability. However, ERL had a most pronounced effect to HEC-1A compared with Ishikawa and KLE in cell viability (HEC-1A 38 %, Ishikawa 78 %, and KLE 72 %, respectively) (Fig. 5b). In the case of TRA treatment (Fig. 5a and b), only Ishikawa cells showed a decrease in ERK 1/2 phosphorylation (*P* < 0.05) and cell viability to 78 % compared with vehicle control (*P* < 0.05).

### Tumor growth inhibition assay following ErbB inhibitor treatment in mice xenograft model

**Fig. 6:**
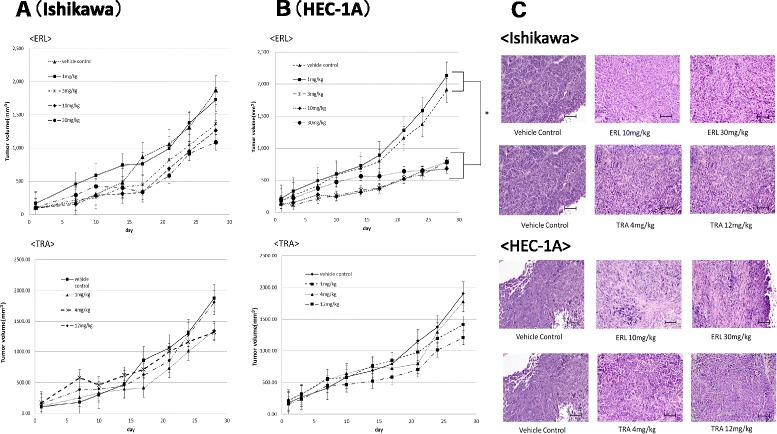
Inhibition of tumor growth by erlotinib (ERL) in vivo. Mice were implanted with Ishikawa (**a**) or HEC-1A (**b**) and treated with ERL or TRA for 28 days. Tumor volume was measured twice a week. Data represent the means ± SEMs of three independent tumor volumes. *, decreased as compared to vehicle control, *P* < 0.05. **c** On day 28 after starting treatment, mice were euthanized and tumor was excised. The tissues were fixed in formalin and embedded in paraffin. Sections were taken from the paraffin-embedded tissue and HE stained. Top and upper middle panels, Ishikawa tumor; lower middle and bottom panels, HEC-1A tumor. Magnification × 200.Bars =1000 μm

Because the in vitro studies were examined for short periods, the long-term effect of either ERL or TRA was studied using an EC xenograft in vivo model. Tumor-bearing mice were treated with either ERL or TRA for 28 days. The results showed that only tumors in HEC-1A xenografted mice treated with ERL at a dose of 3 mg/kg or more (Fig. 6a and b) were reduced, whereas TRA did not induce significant tumor growth inhibition in mice implanted with either HEC-1A or Ishikawa. The resected tumor from the xenograft model stained with HE, suggested that clear fibrosis occurred in HEC-1A tumor treated with ERL (Fig. 6c).

## Discussion

In the present study, we demonstrate that both EGFR mRNA and EGFR protein were highly expressed in low-grade endometrioid carcinoma, but the expression was low in high-grade endometrioid carcinoma. We examined the molecular factors that underlie the variable responsiveness to erlotinib in accordance with the expression levels of both EGFR mRNA and EGFR protein in the endometrial carcinoma cells, using quantitative RT-PCR and IHC. We found that erlotinib, a known potent selective inhibitor of the EGFR tyrosine kinase, significantly inhibits the proliferation of endometrial carcinoma cells, which express high levels of EGFR in xenograft mice models.

The degree of tumor differentiation is one of the prognostic factors in EC; low-grade endometrioid tumors tend not to progress to deep myometrial invasion or spread to distant sites [35]. In contrast, high-grade endometrioid tumor is aggressive and diagnosed at advanced stages, it involves recurrent or metastatic tumors at high rate. On the other hand, overall prognosis for those who are diagnosed with low-grade tumor is positive, although the number of patients with recurrent or metastatic tumors is still large due to the corresponding amount of newly diagnosed type I EC patients [3, 4]. In fact, in our institution, 20.9 % of endometrial cancer patients with low-grade endometrioid histology have been diagnosed as stage III and IV (Table 1). We comprehensively analyzed EGFR and HER2 expression levels in endometrioid carcinoma (Fig. 1), demonstrating that EGFR mRNA and protein were highly expressed in low-grade endometrioid tumor compared to high-grade endometrioid tumor. In contrast, HER2 was not significantly expressed at a varying level in any grade of endometrioid tumor. EGFR mediates the activation of intracellular signaling pathways, such as MAPK-ERK and PI3K-AKT pathways, resulting in enhanced proliferation and cell survival in EC cells. Collectively, these results prompted us to further investigate the significance of EGFR in the proliferation of low-grade endometrioid tumor.

To date, anti-EGFR antibody, anti-EGFR, or dual EGFR/HER2 tyrosine kinase inhibitors have been evaluated across a variety of disease types. For HER2-positive patients with breast cancer, trastuzumab has significantly reduced the rate of recurrence [36]. In the subsequent study [37], lapatinib, the EGFR and HER2 dual kinase inhibitor, displayed significant antiproliferative effects in HER2 overexpressing breast tumor cell lines, suggesting that the EGFR expression level has no association with the sensitivity to lapatinib. In contrast, both EGFR and HER2 expression has been found in patients with non-small-cell lung cancer with poor prognosis [38], erlotinib was beneficial in those patients in an EGFR-dependent way [39]. In this study, we confirmed that the amount of EGFR expression plays an important role in MAPK pathway in endometrial cancer cell (Fig. 3), suggesting that endometrial cancer with highly expressed EGFR is strongly effected by anti-EGFR drugs. Furthermore, the antitumor effects of erlotinib against HEC-1A cells clearly inhibited tumor growth both in vitro (Fig. 5b) and in vivo (Fig. 6b). On the other hand, trastuzumab did not reduce the tumor growth of Ishikawa cells in xenograft mice (Fig. 6a). Taken together, the current data indicate that the expression levels of EGFR is a key factor in the molecular targeted therapy against pathogenic tyrosine kinases in endometrial cancer, and suggest that EGFR inhibitors may be clinically useful for well-defined subgroups of endometrial cancer patients with highly-expressed EGFR.

A phase II study (NCIC IND-148) has been largely considered to have concluded that erlotinib is not a promising agent for recurrent or metastatic EC. However, in that study, tumors were regarded as EGFR positive if tumor cell membranes stained positively with anti-EGFR antibody in IHC in more than 10 % of tumor cells. Thus, we speculate that this clinical study contained large cases of high-grade endometrioid tumors and type II EC, based on our finding that a majority of cell membranes were stained in low-grade endometrioid tumors (Fig. 1).

Patients with risk factors such as tumor grade, deep myometrial invasion, and positive lymph nodes are recommended for systemic chemotherapy, although it is not unanimously accepted. Basic cancer research is conducted to identify the markers that determine patients to chemotherapy regimen according to the responses. In malignant tumors, it is unlikely that one signaling pathway is solely engaged in its aggressive behavior including progression and metastasis. However, the present data demonstrate that erlotinib has efficacy in the treatment of endometrial cancers, which highly express EGFR. We believe that further analysis of the molecular signature of the EC tumors will define patients who can be benefited by erlotinib therapy.

## Conclusions

Type I EC accounting for 80 % of EC is associated with better clinical outcome than type II EC. However, a substantial number of patients have to deal with recurrent tumors due to corresponding amounts of type I EC patients. In this study, we found that EGFR protein was highly expressed in low grade endometrioid carcinoma tissue and that the proliferation of an EC cell line with high EGFR expression was attenuated by erlotinib, an EGFR tyrosine kinase inhibitor. In tumor xenograft mouse models, erlotinib clearly reduced the growth of tumors with high levels of EGFR expression. This agent could be further developed for pre-clinical and clinical studies specific patient subgroups in advanced stage or recurrent EC with high-EGFR expressed tumors.

## Abbreviations

EC: Endometrial carcinoma
EGF: Epidermal growth factor
EGFR: Epidermal growth factor receptor
HER-2: Human epidermal growth factor receptor type2
ERK1/2: Extracellular Signal-regulated Kinase 1/2
MAPK: Mitotic-activated protein kinase
ERL: Erlotinib
TRA: Trastuzumab
IHC: Immunohistochemistry
